# Social Isolation and Mortality in Older Adults in Sweden: A Cohort Study

**DOI:** 10.3389/ijph.2025.1608729

**Published:** 2025-11-13

**Authors:** Senait Wolde, Junmei Miao Jonasson

**Affiliations:** School of Public Health and Community Medicine, Institute of Medicine, Sahlgrenska Academy, University of Gothenburg, Gothenburg, Sweden

**Keywords:** social isolation, all-cause mortality, cause-specific mortality, cohort study, older adults

## Abstract

**Objectives:**

This study examined the association between social isolation and mortality outcomes in a large Swedish cohort.

**Methods:**

A cohort study was conducted among 36,490 men and women aged 56–95 years based on linking the Swedish Mammography Cohort (SMC) and the Cohort of Swedish Men (COSM) with Swedish national registers. Cox regression models were used to estimate associations between social isolation and mortality.

**Results:**

Participants with high social isolation had a significantly higher risk of all-cause mortality compared to those with low social isolation (HR 1.17, 95% CI: 1.09–1.27). This association was observed in both women (HR 1.18, 95% CI: 1.02–1.37) and men (HR 1.15, 95% CI: 1.05–1.27). For cause-specific mortality, social isolation was significantly associated with deaths from ischemic heart disease (HR 1.55, 95% CI: 1.12–2.14) and prostate cancer (HR 1.44, 95% CI: 1.02–2.04).

**Conclusion:**

Our study found a significant association between social isolation and both all-cause and cause-specific mortality, such as ischemic heart disease and prostate cancer, among older adults.

## Introduction

Social isolation is defined as “the objective lack of (or limited) social contact with others,” typically characterized by few social ties, infrequent interactions, or living alone [1, 2]. It is measured using objective indicators—such as number of contacts, frequency of interaction, and network size—that capture the structural aspects of social networks. Although self-reported, these indicators are considered objective as they capture quantifiable characteristics of social connections rather than subjective perceptions. Social isolation is a structural network characteristic of social networks rather than loneliness or perceived support. It is used to assess how the absence of social contacts contributes to mortality risk independently of subjective experiences.

Social isolation differs from loneliness, which is a subjective feeling of being isolated [2]. Social support refers to the resources provided by others in the social network [3]. Although isolation and loneliness often overlap, they are not interchangeable: some individuals may be isolated without feeling lonely, while others may feel lonely despite having many social connections [4].

Social relationships play a crucial role in both physical and mental health by providing emotional and instrumental support (e.g., practical help with daily tasks), shaping health behaviors, buffering stress, and facilitating access to care [5]. Through these mechanisms, social relationships enhance psychological wellbeing, reduce the risk of depression, and contribute to physiological regulation, including immune and neuroendocrine function [5].

Conversely, the absence of social relationships has emerged as a major public health concern. Social isolation has been found to have health effects comparable to alcohol consumption and tobacco use [6–8], and its prevalence increases with age [9]. The mechanisms linking social isolation to mortality can be understood through behavioral, psychological, and physiological pathways. Isolation reduces access to social support and engagement, increasing the likelihood of unhealthy behaviors such as physical inactivity, poor diet, and low adherence to medical care. Physiologically, isolation has been associated with chronic inflammation, immune suppression, and dysregulation of the hypothalamic–pituitary–adrenal axis. Psychosocially, isolation contributes to chronic stress, depression, and diminished self-efficacy and self-esteem. These pathways are illustrated in the social epidemiology framework which situates social networks as upstream social-structural determinants that shape psychosocial processes and, in turn, health outcomes [10]. Within this framework, our study examines social isolation as a structural network characteristic and its association to all-cause and cause-specific mortality among older Swedish adults.

Non-communicable diseases, particularly cancer and cardiovascular disease, are the leading causes of death among older adults [11, 12]. In Europe, Cardiovascular Disease (CVD) remains the top cause of mortality in both men and women, followed by cancer [13–15]. In Sweden, ischemic heart disease (IHD) is the most common cause of death in men and the second most common in women, while Alzheimer’s disease and other dementias are the leading cause of death in women [12, 16]. Regarding cancer, prostate cancer is the leading cause of cancer-related death among men, whereas lung cancer is the leading cause among women, followed by breast cancer [12, 17]. Given the substantial burden of these diseases, identifying factors such as social isolation that may contribute to mortality is critical for developing preventive strategies and reducing premature mortality among older adults.

A growing body of research consistently demonstrates an association between social isolation and a higher risk of mortality in diverse populations. For example, this association was shown in studies of African-Americans aged 20–90 years [18], individuals with obesity in the UK Biobank cohort [19], older adults in Japan and in England [20], and adults over 50 in Mexico [21]. Notably, one recent study found that social isolation increased 20-year mortality risk by more than 15% [22]. Furthermore, findings from the Prospective Urban Rural Epidemiology (PURE) study revealed that social isolation was associated with an increased risk of all-cause mortality among adults aged 35–70 across countries with differing socioeconomic conditions [23].

Research examining cause-specific mortality has shown that socially isolated individuals face a higher risk of death from CVD [24] and cancer [25, 26]. A meta-analysis further demonstrated that social isolation significantly increases the risk of both CVD and cancer, specifically breast cancer [18]. Among individuals already diagnosed with cancer, social isolation has also been linked to increased mortality [27].

Sweden is a particularly relevant context for studying social isolation, as it has one of the highest proportions of older adults living alone globally, a trend that has increased over time [28, 29]. Older adults living alone are at a higher risk of experiencing loneliness and social isolation, and a recent study found that approximately 31% of men and 59% of women aged 75 and older in Sweden live alone [29]. Among this group, 52.6% of men and 73.7% of women reported feeling lonely [30].

A previous Swedish study with a 5-year follow-up found that both social isolation and loneliness were associated with increased mortality risk [26]. However, the study had several limitations: first, social isolation was measured using limited indicators that did not capture the full range of social contacts and activities; second, relatively few participants experienced both high isolation and high loneliness, limiting analysis of their combined effects; and third, reverse causality was a concern, as poor health could lead to social isolation. Unlike prior research, our study addresses these limitations by employing a longer follow-up period of up to 10 years, thereby strengthening causal inference. We also use a broader set of indicators to capture social isolation as a structural network characteristic, providing a more comprehensive assessment of social connections. Moreover, our large population-based cohort increases the statistical power to explore associations with both all-cause and cause-specific mortality.

This study examines the association between social isolation and both all-cause and cause-specific mortality among Swedish older adults aged 56–95 years, by linking comprehensive questionnaire survey data to the Swedish Cause of Death register. By including cause-specific mortality, this study seeks to provide more detailed insights into how social isolation affects health outcomes, extending beyond the scope of previous studies.

## Methods

This study received ethical approval from the Swedish Ethical Review Authority (DNR- 2019-03643).

### Study Design

Cohort study based on linkage of questionnaire data to Swedish national registers.

### Participants

This study was based on the linkage of two population-based Swedish cohorts using Swedish registers: the Swedish Mammography Cohort (SMC), which included women born between 1914 and 1948, and the Cohort of Swedish Men (COSM), which included men born between 1918 and 1952. Both cohorts conducted follow-up surveys in between 2008-2009, which served as the baseline for the present study. Information on education level was obtained from the 1997 SMC survey.

**FIGURE 1:**
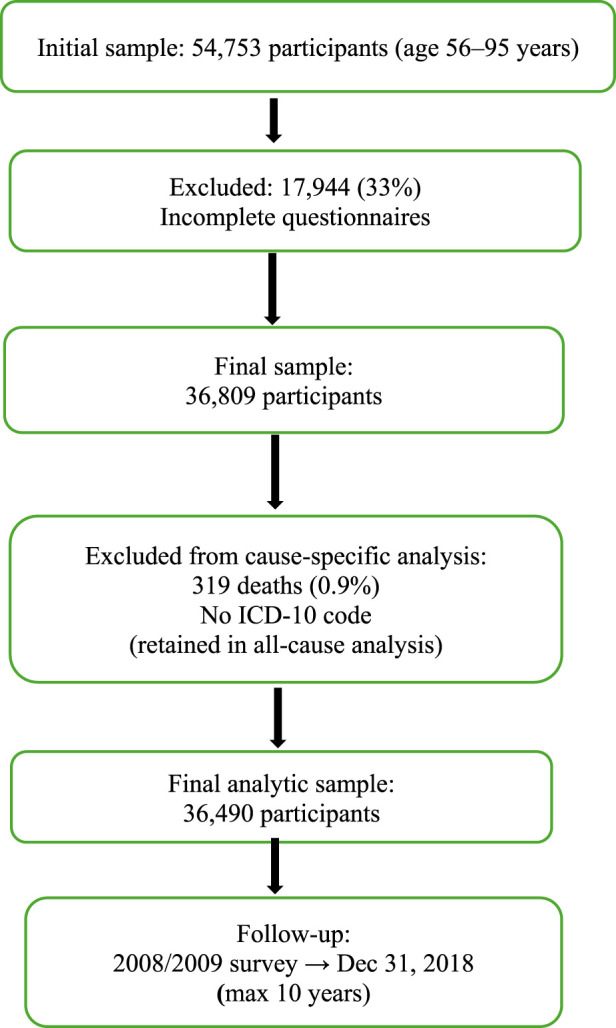
Flowchart on selection of study participants (Sweden, 2008–2018).

The combined registers contained information on 54,753 individuals aged 56–95 years. After data cleaning, 17,944 participants (33%) were excluded from this study due to incomplete questionnaires, yielding a final sample of 36,809. Among these, 319 deaths (0.9%) without an ICD-10 cause-of-death code were excluded from cause-specific analyses but retained in the all-cause mortality analysis, resulting in a final analytic sample of 36,490 participants. Participants were followed from the date of the 2008/2009 survey until death or the end of the follow-up on December 31, 2018, whichever occurred first (maximum follow-up: 10 years). Figure 1 presents a flow diagram of study participants.

**FIGURE 2:**
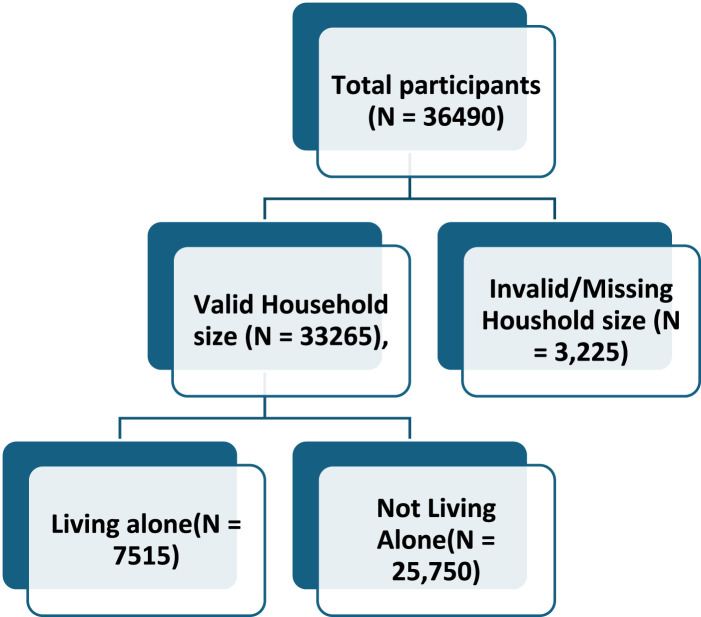
The general information of participants living alone (Sweden, 2008–2018).

Additional exclusions were applied to the household size variable. A total of 3,225 individuals reported household sizes of 0 or ≥15 members, which precluded classification of their living arrangements. To address this, a flag variable was created for a valid household size (1–14 members). Participants with valid data were classified as living alone if they reported exactly one household member and not living alone if they reported two or more household members. Individuals reporting 0 or ≥15 household members (n = 3,225) were excluded from analyses involving the living alone variable to prevent bias. No deaths occurred among the excluded participants. A flowchart of the sample selection process is presented in Figure 2.

### Exposure Variable

The main exposure, social isolation, was assessed using the Lubben Social Network Scale–6 (LSNS-6) [22, 31, 32]. This validated tool consists of six items that assess family and friend networks, including frequency of contact and availability of emotional and practical support. The full scale, including all items, is shown in Appendix 2.

Each item was scored 0 to 5, for a total score of 0–30; a score <12 indicates risk of social isolation [31]. For analysis, participants were categorized into three groups: High risk (0–12 points), Moderate risk (13–18 points), and Low risk (19–30 points). The LSNS-6 demonstrated strong validity, with Cronbach’s α values of 0.90 (family subscale) and 0.95 (friend subscale) in a prior study [31]. In our study population, internal consistency was good (Cronbach’s α = 0.87; average inter-item covariance = 0.69).

### Outcome Variable

The primary outcome variables in this study was all-cause and cause-specific mortality, identified through linkage with the Swedish Causes of Death Register. This register provides complete information on the date and cause of death for all Swedish residents.

The underlying cause of death was classified using ICD-10 codes [33]. ICD-10 categories with very few cases were grouped as “Less Common Causes” to maintain sufficient analytical power.

### Covariates

Based on established associations with social isolation and mortality [34], covariates in this study included sociodemographic factors (age, sex, education, employment status, living conditions, living alone), lifestyle factors (smoking, alcohol consumption, physical activity), and health-related factors (comorbidity, self-rated health, physical condition, self-reported stress). Variable categorization was based on cohort data.

Participants self-reported their general health (“How are you currently feeling in general?”) and physical status (“How is your physical condition?”), with responses ranging from *very good* to *very bad*. Participants were categorized by age (56–65, 56–75, 76–85, 86–95) and level of education (Primary school, High school, and University). Employment status at baseline was recorded as full-time, part-time, not working, disabled, or retired; for regression analyses, they were categorized as retired or non-retired (full-time, part-time, or disability). Living conditions (e.g., home ownership, assisted living) and living alone (yes/no, derived from household size) were also assessed.

The comorbidity index was calculated from self-reported chronic conditions, including hypertension, cholesterol, angina, heart failure, asthma, depression, and diabetes, and categorized into three groups: no comorbidity, one comorbidity, or two or more comorbidities.

Alcohol consumption was assessed using two items: “Do you currently drink alcohol?” (Yes/No) and “Have you ever had alcohol?” (*never* or *stopped drinking*). Only five individuals reported being former drinkers, and they were thus combined with never-drinkers; participants were therefore classified as current drinkers or non-drinkers. Smoking status was based on the question “Have you ever smoked cigarettes regularly (more than five per week)?” with responses *never*, *current*, or *former*, consistent with prior analyses in the SMC and COSM cohorts [35].

Physical activity, defined as activity causing shortness of breath for more than 2 hours per week, was categorized as yes or no. Chronic stress was assessed with the question, “Have you experienced constant stress in the past year in private life?” (examples included tension, irritability, anxiety, sleep difficulties, sadness, or powerlessness), and the response was categorized as yes or no [36].

### Statistical Analyses

Both descriptive and inferential statistical analyses were performed. Inferential statistics were performed using Cox proportional hazards models to estimate hazard ratios (HRs) for the association between social isolation and mortality outcomes. We examined both all-cause mortality (stratified by sex) and cause-specific mortality using the underlying cause of death (ICD-10). Five models were created. Model 1 was adjusted for age and sex; Model 2 additionally adjusted for lifestyle factors such as smoking status, alcohol consumption, and physical activity; Model 3 further adjusted for comorbidity. Model 4 included stress in private (yes/no), and Model 5 was additionally adjusted for socioeconomic factors (education, employment, living conditions, and living alone).

In addition to the primary cause-specific analyses, we examined contributory causes of death listed on death certificates to capture comorbid conditions. Deaths due to cardiovascular disease (CVD), cancer, diabetes, dementia, and social isolation were flagged across all cause-of-death fields, and binary indicators were created for each condition. Cross-tabulations were used to describe the distribution of primary causes among individuals with these secondary conditions, for example, cases in which CVD was a contributory cause but not the primary cause. Deaths with valid ICD-10 codes that did not match predefined categories (e.g., CVD, cancer, diabetes, dementia) were grouped as “valid but not classified,” a heterogeneous category containing too few cases to analyze separately.

## Results

The participants had an average age of 68.75 years (SD = 7.85), ranging from 56 to 95 years, and just over half (55.91%) were men, while just under half were women (44.09%). The mean follow-up time was 10.17 years (SD = 2.05). Chronic stress in private life was reported by a small proportion (5.89%) of the sample. Nearly all participants (99.24%) lived in their own homes, with very few residing in assisted living or retirement facilities. Approximately one-quarter of the participants lived alone. Table 1 presents baseline characteristics of the study population.

**TABLE 1:** Baseline characteristics of study population (Sweden, 2008–2018).

Variables	N (%)	Variables	N (%)	Variables	N (%)
Age in Years [Mean ± SD]	68.75 ± 7.85	Current Health Status		Smoking regularly	
Age in Category		Very Good	6,835 (18.73)	Never smoked	18,523 (50.76)
56–65	15,237 (41.76)	Good	20,429 (55.99)	Used to smoke	14,588 (39.98)
56–75	13,473 (36.92)	Okay	8,404 (23.03)	Currently smoking	3,379 (9.26)
76–85	6,702 (18.37)	Bad	751 (2.06)	Drinking status	
86–95	1,078 (2.95)	Very Bad	71 (0.19)	Not drinking	4,372 (11.98)
Gender		Current physical status		Currently drinking	32,118 (88.02)
Female	16,090 (44.09)	Very Good	3,315 (9.08)	Physical activity	
Male	20,400 (55.91)	Good	16,657 (45.65)	No	16,248 (44.53)
Employment		Okay	13,725 (37.61)	Yes	20,242 (55.47)
Full-time	8,260 (22.64)	Bad	2,528 (6.93)	Living alone	
Part-time	2,705 (7.41)	Very Bad	265 (0.73)	Yes	7,515 (22.59)
Not working	778 (2.13)	Comorbidity category		No	25,750 (77.14)
Disability	1,542 (4.23)	No comorbidity	14,398 (39.46)	Valid Household	
Retired	23,205 (63.59)	At least one comorbidity	11,527 (31.59)	Yes	33,365 (91.16)
Current living condition		At least two Comorbidities	10,565 (28.95)	No	3,225 (8.84)
Home	36,213 (99.24)	Pets in the household		Chronic stress in private	
Assisted living facility	156 (0.43)	No	27,919 (76.51)		
Retirement home	121 (0.33)	Yes	8,571 (23.49)	Yes	2,148 (5.89)
Education		Activity in any clubs		No	34,342 (94.11)
Primary school	10,947 (30.00)	No	18,184 (49.83)	Social Isolation category using LSNS-6	
High school	17,183 (47.09)	Yes	18,306 (50.17)	Low risk of Social Isolation	7,320 (20.06)
University	8,360 (22.91)			Moderate risk of Social Isolation	14,272 (39.11)
				High risk of Social Isolation	14,898 (40.83)

Nearly half of the participants did not engage in physical activity, while 55.47% reported some form of exercise. A large majority (88.02%) were current drinkers, while about half (50.76%) had never smoked, more than a third (39.98%) were former smokers, and nearly one in ten (9.26%) were current smokers. Social relationships, a key focus of the study, were assessed using the LSNS-6 scale to measure social isolation. Based on this measure, about the same proportion of participants were categorized as being at high risk (40.83%) and moderate risk (39.11%) of social isolation, while about one in five (20.06%) at were categorized as low risk. Club or social activity participation was relatively low, with about half (49.82%) of participants not engaged in such activities, highlighting the potential difficulties they may face in maintaining strong social networks.

During the follow-up period, about one in six (17.43%) participants had died, while the majority (82.57%) of participants were censored. The overall mortality rate was 17.14 deaths per 1,000 person-years. Table 2 presents the primary causes of death. CVD and cancer were the most common causes of death. Within CVD, acute myocardial infarction (AMI) was a leading cause, with 467 deaths and a mortality rate of 173.24 per 1,000 person-years. Ischemic heart disease (IHD) followed with 492 deaths and a mortality rate of 155.61 per 1,000 person-years. Among cancer-related deaths, breast cancer was a significant cause of death among women, with 102 deaths (1.60%) and a mortality rate of 177.04 per 1,000 person-years. Prostate cancer was a major cause among men, with 336 deaths (5.28%) and a mortality rate of 162.58 per 1,000 person-years.

**TABLE 2:** Distribution of primary causes of death and mortality rates per 1,000 person-years in the study population (Sweden, 2008–2018).

Cause specific	ICD-10 codes	Frequency and %	Mortality rate per 1,000 person-years	Cause specific and ICD-10 codes	ICD-10 codes	Frequency and %	Mortality rate per 1,000 person-years
Acute Myocardial Infarction (AMI)	I20-I22	467 (7.34)	173.24	Pancreatic Cancer	C25	191 (3.00)	164.7
Ischemic Heart Disease (IHD)	I20-I25	492 (7.73)	155.61	Gynecological Cancer Endometrial and Ovary Cancer	(C56) and (C54)	82 (1.29)	177.1
Heart Failure (HF)	I50	215 (3.38)	157.68	Cancer (Other)	Other C codes	803 (12.62)	167.94
Stroke	I60-I64	282 (4.43)	163.55	Unspecified Cancer	C76, C80, C97	100 (1.57)	166.32
Other Cardiovascular Disease	I00-I99 excluding above	849 (13.34)	154.76	Chronic Respiratory Disease	J40-J47	186 (2.92)	156.28
Lung Cancer	C33-C34	326 (5.12)	171.06	Alzheimer and Dementia	G30, F01, F03	304 (4.78)	132.71
Breast Cancer	C50-C509	102 (1.60)	177.04	Ill Defined	R99	60 (0.94)	163.65
Colorectal Cancer	C18-C20	239 (3.76)	168.94	Other or Less Common Cause Diabetes, Liver Disease, Chronic Kidney Disease, Undetermined, Unspecified Exposure	(E10-E14)	150 (2.36)	155.73
Prostate Cancer	C61	336 (5.28)	162.58	Unknown - Valid but not classified Valid ICD not classified	(J189,W19,J841,A419,G20,B99,G122,E140)	1,178 (18.52)	153.92

### All-Cause Mortality

Table 3 presents the HRs and 95% CI from Cox regression for those with high or moderate risk, compared with low risk, of social isolation. In Model 1, which adjusts for age and sex, participants with a high social isolation had a significantly increased hazard of death (HR = 1.33; 95% CI: 1.24–1.43, p < 0.001) compared to the reference group with low social isolation. This suggests participants with high social isolation had a 33% higher hazard of death. Participants with moderate social isolation also had a higher hazard of death (HR 1.10; 95% CI: 1.02–1.19, p < 0.05).

**TABLE 3:** Hazard ratio and 95% Confidence Intervals for mortality in groups with high or moderate risk of social isolation compared with low risk of social isolation in the total study population (Sweden, 2008–2018).

Social category	Model 1	Model 2	Model 3	Model 4	Model 5
Low risk of social isolation	Ref	Ref	Ref	Ref	Ref
High risk of social isolation	1.33 (1.24–1.43)***	1.25 (1.16–1.34)***	1.22 (1.13–1.31)***	1.21 (1.12–1.30)***	1.17 (1.09–1.27)***
Moderate risk of social isolation	1.10 (1.02–1.19)*	1.09 (1.01–1.17)*	1.08 (1.00–1.16)	1.07 (0.99–1.16)	1.07 (0.99–1.16)

HR, hazar ratio; SD, Standard Deviation *P < .05; **P ≤ .005; ***P ≤ .001.

Model 1: adjusted for age and sex (as categorical variable).

Model 2: adjusted for age, sex (as categorical variable), lifestyle factors (smoking, alcohol drinking, physical activity).

Model 3: adjusted for age, sex (as categorical variable), lifestyle factors (smoking, alcohol drinking, physical activity); comorbidity index (as categorical variable).

Model 4: adjusted for age, sex (as categorical variable), lifestyle factors (smoking, alcohol drinking, physical activity); comorbidity index (as categorical variable); chronic stress in private (categorical variable).

Model 5: adjusted for age, sex (as categorical variable), lifestyle factors (smoking, alcohol drinking, physical activity); comorbidity index (as categorical variable); chronic stress in private (categorical variable); socioeconomic factors (education, employment, living condition, and living alone.

In Model 2, which additionally adjusted for lifestyle factors (smoking, alcohol consumption, and physical activity), high social isolation remained significantly associated with increased mortality hazards (HR = 1.25 (95% CI: 1.16–1.34, p < 0.001). Moderate social isolation was also associated with mortality in this model (HR = 1.09 (95% CI: 1.01–1.17, p < 0.05).

In Model 3, which includes further adjustments for comorbidity status, high social isolation remained significantly associated with mortality (HR = 1.22 (95% CI: 1.13–1.31, p < 0.001), indicating that those with high social isolation had a 22% higher hazard of mortality. Participants in the moderate social isolation showed a borderline association (HR = 1.08 (95% CI: 1.00–1.16).

In Model 4, adjusted for chronic stress in private, high social isolation continued to predict mortality (HR = 1.21, 95% CI: 1.12–1.30, *p* < 0.001), while moderate social isolation was not significantly associated with mortality (HR = 1.06, 95% CI: 0.98–1.14, *p* = 0.19).

In Model 5, which included adjustments for all variables in previous models and additional socioeconomic factors (education, employment, living conditions, and living alone), high social isolation was still significantly associated with increased mortality hazards (HR = 1.17, 95% CI: 1.09–1.27, *p* < 0.001). Moderate social isolation again showed no association with mortality (HR = 1.05, 95% CI: 0.97–1.13, *p* = 0.23.

### All-Cause Mortality Stratified by Sex

Table 4 presents the HRs and 95% CI from Cox regression analyses stratified by sex. Among women, high social isolation was significantly associated with increased mortality hazards in Model 1 (HR = 1.36, 95% CI: 1.20–1.54). This association remained significant in the fully adjusted Model 5 (HR = 1.18, 95% CI: 1.02–1.37, p < 0.05). Moderate social isolation was not significantly associated with mortality among women (HR = 1.06, 95% CI: 0.96–1.16, *p* = 0.24). Among men, high social isolation was also associated with higher mortality in Model 1 (HR = 1.31, 95% CI: 1.19–1.44). In Model 5, the association persisted (HR = 1.15, 95% CI: 1.05–1.27, p < 0.01). Moderate social isolation was not significantly associated with mortality in men in model 5 (HR = 1.10, 95% CI: 1.00–1.22, p = 0.051), though the estimate suggested a possible relationship.

**TABLE 4:** Hazard ratios and 95% Confidence Intervals for mortality in groups with high or moderate risk of social isolation compared with low risk of social isolation in women and men, respectively (Sweden, 2008–2018).

	Women					Men				
Social category	Model 1	Model 2	Model 3	Model 4	Model 5	Model 1	Model 2	Model 3	Model 4	Model 5
Low risk of social isolation	Ref	Ref	Ref	Ref	Ref	Ref	Ref	Ref	Ref	Ref
Moderate risk of social isolation	1.05 (0.92–1.19)	1.01 (0.89–1.15)	1.01 (0.89–1.15)	1.00 (0.88–1.15)	1.01 (0.87–1.17)	1.14 (1.03–1.25)**	1.13 (1.03–1.25)*	1.12 (1.02–1.23)*	1.11 (1.01–1.23)*	1.10 (1.00–1.22)
High risk of social isolation	1.36 (1.20–1.54)***	1.25 (1.10–1.41)***	1.22 (1.08–1.39) ***	1.20 (1–05-1.37)**	1.18 (1.02–1.37)*	1.31 (1.19–1.44)***	1.25 (1.14–1.37)***	1.21 (1.10–1.33)***	1.20 (1.10–1.32)***	1.15 (1.05–1.27)**

HR, hazar ratio; SD, Standard Deviation *P < .05; **P ≤ .005; ***P ≤ .001.

Women: Female-specific Analysis and Men: Male-specific Analysis.

Model 1: Adjusted for age.

Model 2: Adjusted for age and lifestyle factors (e.g., smoking, alcohol consumption, physical activity).

Model 3: Adjusted for age, lifestyle factors, and comorbidity index.

Model 4: adjusted for age, sex (as categorical variable), lifestyle factors (smoking, alcohol drinking, physical activity); comorbidity index (as categorical variable); chronic stress in private (categorical variable).

Model 5: adjusted for age, sex (as categorical variable), lifestyle factors (smoking, alcohol drinking, physical activity); comorbidity index (as categorical variable); chronic stress in private (categorical variable); socioeconomic factors (education, employment, living condition, and living alone.

### Cause-Specific Mortality

Table 5 displays the HR and 95% CI of the association between cause-specific mortality and social isolation. For cardiovascular categories, high social isolation was significantly associated with ischemic heart disease (IHD) mortality in the fully adjusted Model 5 (Model 5: HR = 1.55, 95% CI: 1.12–2.14, p < 0.01). Moderate social isolation also showed a borderline association with IHD mortality in Model 5 (HR = 1.40, 95% CI: 1.00–1.95, p = 0.049). However, no significant associations were observed for acute myocardial infarction, heart failure, stroke, or other cardiovascular causes in the fully adjusted model. Among cancer types, the pattern varied. High social isolation was significantly associated with prostatic cancer mortality in Model 5 (HR of 1.44 (95% CI: 1.02–2.04, p < 0.05). However, in breast cancer, the result revealed no significant association in any of the five models.

**TABLE 5:** Hazard ratio and 95% Confidence Intervals for cause-specific mortality in groups with high or moderate risk of social isolation compared with low risk of social isolation in the total study population (Sweden, 2008–2018).

Cause of death	Social category	Model 1	Model 2	Model 3	Model 4	Model 5
AMI	Low risk of social isolation	Ref	Ref	Ref	Ref	Ref
	Moderate risk of social isolation	0.86 (0.63–1.16)	0.84 (0.62–1.15)	0.86 (0.63–1.17)	0.88 (0.65–1.21)	0.81 (0.57–1.14)
	High risk of social isolation	1.01 (0.75–1.35)	0.99 (0.74–1.33)	0.99 (0.74–1.34)	1.02 (0.76–1.37)	0.94 (0.67–1.31)
IHD	Low risk of social isolation	Ref	Ref	Ref	Ref	Ref
	Moderate risk of social isolation	1.19 (0.88–1.61)	1.23 (0.91–1.67)	1.25 (0.92–1.70)	1.25 (0.92–1.70)	1.40(1.00–1.95)*
	High risk of social isolation	1.35(1.01–1.81)*	1.37(1.02–1.84)*	1.40(1.04–1.87)*	1.39(1.04–1.87)*	1.55(1.12–2.14)**
HF	Low risk of social isolation	Ref	Ref	Ref	Ref	Ref
	Moderate risk of social isolation	1.11 (0.69–1.79)	1.01 (0.63–1.64)	1.00 (0.62–1.62)	1.02 (0.63–1.64)	1.00 (0.63–1.70)
	High risk of social isolation	1.22 (0.79–1.88)	1.08 (0.69–1.67)	1.05 (0.68–1.63)	1.07 (0.69–1.67)	0.94 (0.58–1.51)
STROKE	Low risk of social isolation	Ref	Ref	Ref	Ref	Ref
	Moderate risk of social isolation	0.77 (0.52–1.15)	0.82 (1.54–1.24)	0.79 (0.52–1.20)	0.74 (0.53–1.20)	0.81 (0.51–1.27)
	High risk of social isolation	0.87 (0.59–1.27)	0.90 (0.61–1.32)	0.86 (0.58–1.27)	0.88 (0.59–1.30)	0.85 (0.55–1.30)
Other cardiovascular	Low risk of social isolation	Ref	Ref	Ref	Ref	Ref
	Moderate risk of social isolation	0.99 (0.78–1.24)	0.99 (0.79–1.25)	1.00 (0.79–1.26)	1.00 (0.78–1.25)	1.04 (0.81–1.33)
	High risk of social isolation	1.14 (0.92–1.42)	1.15 (0.92–1.43)	1.15 (0.92–1.43)	1.15 (0.92–1.43)	1.19 (0.94–1.51)
Breast Cancer	Low risk of social isolation	Ref	Ref	Ref	Ref	Ref
	Moderate risk of social isolation	0.90 (0.54–1.52)	0.93 (0.55–1.57)	0.92 (0.54–1.56)	0.92 (0.54–1.59)	0.95 (0.44–1.63)
	High risk of social isolation	1.11 (0.64–1.92)	1.02 (0.58–1.81)	1.01 (0.57–1.80)	1.01 (0.56–1.80)	1.06 (0.50–2.26)
Prostatic Cancer	Low risk of social isolation	Ref	Ref	Ref	Ref	Ref
	Moderate risk of social isolation	1.17 (0.85–1.61)	1.18 (0.85–1.63)	1.14 (0.82–1.58)	1.14 (0.82–1.59)	1.19 (0.84–1.68)
	High risk of social isolation	1.32 (0.96–1.81)	1.33 (0.98–1.83)	1.29 (0.94–1.78)	1.29 (0.94–1.79)	1.44(1.02–2.04)*

HR, hazar ratio; SD, Standard Deviation *P < .05; **P ≤ .005; ***P ≤ .001. Bold values indicate statistically significant hazard ratios (p < 0.05).

Model 1: adjusted for age and sex (as categorical variables).

Model 2: adjusted for age, sex (as categorical variable), lifestyle factors (smoking, alcohol drinking, physical activity).

Model 3: adjusted for age, sex (as a categorical variable), lifestyle factors (smoking, alcohol drinking, physical activity), and comorbidity index (as a categorical variable).

Model 4: adjusted for age, sex (as categorical variable), lifestyle factors (smoking, alcohol drinking, physical activity); comorbidity index (as categorical variable); chronic stress in private (categorical variable).

Model 5: adjusted for age, sex (as categorical variable), lifestyle factors (smoking, alcohol drinking, physical activity); comorbidity index (as categorical variable); chronic stress in private (categorical variable); socioeconomic factors (education, employment, living condition, and living alone).

Mortality among individuals with secondary conditions was highly heterogeneous (see Appendix 1). In the cardiovascular secondary group, cardiovascular events remained the predominant cause of death; however, a substantial proportion of deaths were attributable to non-cardiovascular causes, such as various cancers, chronic respiratory disease, and Alzheimer’s/dementia. Nearly one in six (16%) of deaths were classified as “valid but not classified.” Similarly, in the cancer secondary group, cancer-related deaths dominated, but cardiovascular and other non-neoplastic causes also contributed meaningfully to overall mortality.

## Discussion

Our study found a significant association between social isolation and both all-cause and cause-specific mortality among older adults in Sweden. Among men, both moderate and high social isolation were associated with increased mortality hazards, whereas in women, the association was only seen for high social isolation.

These findings align with previous research showing an association between social isolation and increased all-cause mortality in older adults aged 69 years and over in Sweden [26]. Living alone, often referred to as a proxy indicator of social isolation [37], is common in Sweden and has been significantly linked to higher mortality among older adults in Västerbotten County [38]. Studies from other settings showed a similar association [19–21, 23], while higher levels of loneliness have been linked to reduced social support and increased social isolation [39]. Meta-analyses further confirm that social isolation is associated with an elevated risk of mortality [7, 40]. Our findings confirm that social isolation is a predictor of mortality, comparable to other well-established risk factors such as smoking [8].

In the current study, we did not examine potential pathways from social isolation to mortality. However, several mechanisms have been proposed that may explain these relationships. Social isolation has been linked to chronic stress and inflammation [41–43], accelerated aging [44], unhealthy behaviors such as physical inactivity and poor diet [45–47], as well as poorer physical and mental health outcomes [48]. Together, these factors may contribute to the elevated risk of both all-cause and cause-specific mortality observed among socially isolated older adults.

In our analysis, high levels of social isolation were significantly linked to an increased risk of mortality from IHD. This observation is consistent with findings from a 17-year follow-up study of adults aged 18–64 [49] and longitudinal research showing that social isolation significantly heightens the risk of mortality among patients with IHD [50]. Stress-related physiological mechanisms likely play a role. Persistent activation of the hypothalamic–pituitary–adrenal axis and sympathetic nervous system elevates cortisol and catecholamines, which in turn promote endothelial dysfunction, vasoconstriction, platelet activation, reduced heart rate variability, and hypertension—factors that contribute to atherosclerosis formation and precipitate acute coronary events. [49, 51, 52]. Beyond to stress physiology, immune dysregulation may also play a role. Social isolation has been linked to higher levels of circulating Interleukin-6 (IL-6), CRP, and soluble urokinase plasminogen activator receptor (suPAR) [53, 54]. These inflammatory markers accelerate plaque formation and are strongly linked to cardiovascular mortality. In older adults, social isolation correlates with higher levels of IL-6 and CRP values, with elevated IL-6 in particular linked to increased cardiovascular mortality [55, 56].

We found that social isolation was associated with a higher risk of mortality from prostate cancer, corroborating findings from longitudinal cohort studies [57]. Previous research indicates that a lack of social support substantially influences prostate cancer stage at diagnosis and survival [58]. Psychosocial factors such as stigma, relational difficulties, and reduced social support may also delay help-seeking and reduce treatment adherence, ultimately leading to poorer outcomes [21, 59, 60]. In line with these findings, a Swedish cohort study reported that men with higher perceived stress not only had a significantly increased risk of prostate cancer–specific mortality (HR = 1.66; 95% CI: 1.05–2.63), but also reported fewer confidants and felt less able to share their problems with partners, family, and friends [61]. Beyond these psychosocial mechanisms, biological pathways may also be important: chronic stress-related activation of the hypothalamic–pituitary–adrenal axis and the autonomic nervous system may promote angiogenesis (new blood vessel formation) and tumor growth, thereby accelerating cancer progression and mortality [61, 62].

In the case of breast cancer, our study did not identify a significant relationship between social isolation and breast cancer mortality, consistent with research conducted in the United States [59]. This may be explained by high participation in the mammography screening program in Sweden [63], which effectively reduces mortality from breast cancer through early detection and treatment [64]. It is important to note that a Swedish study reported lower mammography screening participation among socially isolated women, highlighting potential disparities in access and utilization of preventive services [65]. Moreover, a large pooled cohort after the Breast Cancer Pooling Project revealed that socially isolated women faced a higher risk of breast cancer recurrence and breast cancer-specific mortality [66]. This discrepancy may reflect differences in study populations, the measurement of social isolation, or statistical power. While we did not investigate these specific sources of variation, they are important considerations when comparing results across studies.

Although social isolation and mortality have been studied in Sweden, evidence remains limited, particularly regarding cause-specific mortality. Our study addresses this gap by using a large sample size and an extended follow-up period, enabling a more thorough assessment of mortality risk over time while reducing potential bias.

This study also has limitations. First, exposure variables were self-reported, which may have introduced reporting bias. Second, social isolation was assessed only at baseline and did not account for changes over time. Future research should use repeated measures to capture the dynamic nature of social relationships. Third, 17,944 participants (33% of the initial sample) were excluded due to incomplete covariate data, raising the possibility of selection bias. Fourth, we did not include certain variables, such as club participation or pet ownership, which may plausibly influence social relationships and health but were not consistently measured. Fifth, we did not stratify by age or retirement status, which may influence patterns of social isolation and mortality. Finally, our analysis was limited to broad categories of cause-specific mortality, and the findings may not generalize to other causes of death or to populations outside Sweden.

In conclusion, social isolation was associated with all-cause mortality as well as cause-specific mortality from IHD in both sexes and prostate cancer in men, while no association was found for breast cancer mortality among older adults in Sweden. These findings highlight the urgent need for public health strategies that foster social engagement, particularly among older adults. Globally, policies addressing social isolation in aging populations should be prioritized, with an emphasis on fostering social integration through community-based programs. Further research is needed to clarify the complex mechanisms by which social isolation influences mortality and to inform the development of targeted interventions that can mitigate its adverse effects.
