# Supplementary material for: Social Isolation and Mortality in Older Adults in Sweden: A Cohort Study
Source: Int J Public Health. 2025 Nov 13;70:1608729. doi: 10.3389/ijph.2025.1608729 (PMC12658459; doi:10.3389/ijph.2025.1608729)
Supplement: Supplementary file 1 [file Supplementaryfile1.pdf]

Appendix 1. The relationship between the primary cause and the secondary cause of mortalities (Sweden, 2008-2018)

Cause specific	Frequency & % among deaths with CVD SECONDARY	DIABETES SECONDARY	CANCER SECONDARY	Dementia and Alzheimer secondary
AMI	467(12.38)	72(10.93)	26(1.05)	20(3.01)
IHD	492(13.04)	91(13.81)	44(1.77)	45(6.77)
HF	215(5.70)	20(3.03)	26(1.05)	29(4.36)
STROKE	282(7.48)	26(3.95)	18(0.73)	18(2.71)
OTHER CVD	849(22.51)	99(15.02)	67(2.70)	100(15.04)
LUNG CANCER	85(2.25)	15(2.28)	326(13.15)	2(0.30)
BREAST CANCER	28(0.74)	3(0.46)	102(4.11)	1(0.15)
COLORECTAL CANCER	43(1.14)	12(1.82)	239(9.64)	5(0.75)
PROSTATIC CANCER	96(2.55)	16(2.43)	336(13.55)	9(1.35)
PANCREATIC CANCER	36(0.95)	10(1.52)	191(7.70)	1(0.15)
GYNECOLOGIC CANCER	12(0.32)	3(0.46)	82(3.31)	1(0.15)
CANCER OTHER	179(4.75)	33(5.01)	803(32.39)	14(2.11)
UNSPECIFIED CANCER	30(0.80)	10(1.52)	100(4.03)	5(0.75)
CHRONIC REPIRATORY	127(3.37)	7(1.06)	20(0.81)	10(1.50)
ALZHIEMER AND DEMENTIA	128(3.39)	23(3.49)	19(0.77)	20(3.01)

LL DEFINED	-	-	-	-
OTHER OR LESS COMMON CAUSE	98(2.60)	48(7.28)	13(0.52)	12(1.80)
VALID BIT NOT CLASSIFIED	605(16.04)	171(25.95)	76(2.70)	89(1.38)

Appendix 2 FAMILY, FRIENDS, AND ACQUAINTANCES(Social relationship questions)

1. How many people, with the same interests as you, do you know and have contact with? Both at work and in spare time

None 1-2 3-5 6-10 11-15 More than 15

2. How many people, that you know well, do you meet or talk with during a normal week? Do not count those that you run into unexpectedly.

None 1-2 3-5 6-10 11-15 More than 15

3. How many friends do you have that can come to your home anytime and feel at home? They would not care if it was untidy or if you were about to eat. Do not count close relatives.

None 1-2 3-5 6-10 11-15 More than 15

4. How many are there, in your family or among your friends who you can talk openly with?

None 1-2 3-5 6-10 11-15 More than 15

5. How many people are there in your environment who you can easily ask for things? People who know you so well that you can borrow tools or kitchen things?

None 1-2 3-5 6-10 11-15 More than 15

6. Apart from those at home, how many are there that you can turn to if you are in difficulty? Someone who you can easily meet and who you trust and can really help you when you are experiencing difficulties?

None 1-2 3-5 6-10 11-15 More than 15
